# Associations between serum levels of insulin-like growth factor-I and bone mineral acquisition in pubertal children: a 3-year follow-up study in Hamamatsu, Japan

**DOI:** 10.1186/s40101-019-0210-5

**Published:** 2019-12-05

**Authors:** Katsuyasu Kouda, Masayuki Iki, Kumiko Ohara, Harunobu Nakamura, Yuki Fujita, Toshimasa Nishiyama

**Affiliations:** 1 Department of Hygiene and Public Health, Kansai Medical University, 2-5-1 Shin-machi, Hirakata, Osaka 573-1010, Japan; 2 Department of Public Health, Kindai University Faculty of Medicine, 377-2 Oono-Higashi, Osaka-Sayama, Osaka 589-8511, Japan; 3 Department of Health Promotion and Education, Graduate School of Human Development and Environment, Kobe University, 3-11 Tsurukabuto, Nada, Kobe, Hyogo 657-8501, Japan

**Keywords:** Bone development, Densitometry, General population, Somatomedins

## Abstract

**Background:**

Epidemiological data regarding the association between serum levels of IGF-I and bone mineral acquisition during childhood are scarce. Here, we investigated the association between serum levels of IGF-I and bone status during puberty.

**Methods:**

We analyzed prospective 3-year follow-up data of 254 community-dwelling children who completed both baselines (at age 11.2 years) and follow-up (at age 14.2 years) surveys in Hamamatsu, Japan. Total body (TB) bone area and bone mineral parameters were assessed using dual-energy X-ray absorptiometry.

**Results:**

During the 3-year follow-up period, there were significant (*P* < 0.05) increases in total body less head (TBLH) areal bone mineral density (aBMD), TBLH bone mineral content (BMC), and TB bone area, and a significant decrease in TB bone mineral apparent density (BMAD, volumetric bone mineral density, vBMD). IGF-I levels showed significant positive relationships with TBLH BMC and TBLH aBMD at both baseline and follow-up. TBLH aBMD in boys and TB BMAD in girls at follow-up showed significant increases from the lowest to highest quartiles of baseline IGF-I levels after adjusting for confounding factors. Similarly, changes in TBLH aBMD in boys and TB BMAD in girls during the 3-year follow-up period showed significant increases from the lowest to highest quartiles of baseline IGF-I levels after adjusting for confounding factors.

**Conclusions:**

These results suggest that pubertal children with high levels of serum IGF-I tended to have high bone mineral acquisition later on.

## Background

Skeletal mass and density at multiple sites, including the whole body, accumulate for the most part relatively early in life [1]. Bone mass content increases mainly during the first 3 years of life and during the pubertal growth spurt [2]. A longitudinal study reported that adolescence is clearly a critical time for bone mineralization because 26% of adult calcium is accumulated in the 2 years of peak skeletal growth [3]. Bone status during childhood, when peak bone mass is accumulated, is indicative of bone status in adulthood [4].

Circulating insulin-like growth factor-I (IGF-I) is a mediator of growth hormone and appears to have a direct impact on bone growth. The liver is the major source of IGF-I, where approximately 75% of circulating IGF-I originates [5]. In addition to circulating IGF-I, most tissues express IGF-I locally [5]. Bone growth relies not only on circulating IGF-I in blood [6], but also autocrine/paracrine IGF-I in tissues [7]. Direct evidence for the importance of the autocrine/paracrine role of IGF-I in body growth has been reported in animal models [7]. According to some experimental animal studies, hepatic IGF-I in the blood is not required for postnatal body growth [7, 8] and liver-derived circulating IGF-I exerts only a small effect on bone growth in mice [9]. In contrast, one experimental animal study reported that a threshold level of circulating IGF-I is necessary for normal bone growth [10]. Further characterization of the role of circulating IGF-I in bone acquisition in an epidemiological human study may have important implications for our understanding of skeletal growth during childhood [10].

However, information from epidemiological studies regarding the association between serum levels of IGF-I and bone mineral acquisition during childhood is scarce. To date, only two prospective studies have investigated the effect of IGF-I on bone mineral accrual in healthy children. The first, a longitudinal study targeting healthy 12-year-old girls (*n* = 37), found no association between serum levels of IGF-I and gain in bone mass during an 18-month follow-up period [11]. The second, a prospective study targeting community-dwelling females (4- to 8-years-old at baseline, *n* = 76), found a significant relationship between changes in IGF-I and changes in bone mineral parameters [12]. In the present 3-year follow-up study, which had a relatively large sample size (*n* = 254), we investigated associations between serum levels of IGF-I at age 11 years and bone mineral parameters such as bone area (cm^2^), bone mineral content (BMC, g), areal bone mineral density (aBMD, g/cm^2^), and volumetric bone mineral density (vBMD, g/cm^3^) from age 11 to 14 years.

## Methods

### Subjects

The source population from which our subjects were selected consisted of all fifth-grade children registered in Aritama Elementary School and Sekishi Elementary School in Hamamatsu, Japan. There were 521 children (268 boys and 253 girls) in the two schools in November/December of 2010 and 2011 when the baseline survey was conducted, as previously described [13]. Since there were no other elementary schools in the school district, most children aged 11 years were enrolled in the two schools. Moreover, most children in this area went on to Sekishi Junior High School, the only junior high school in the area. After 36 months, a follow-up survey was conducted at this junior high school in December 2013 and 2014. Of the source population, we obtained complete baseline data for 408 children (202 boys and 206 girls) and complete follow-up data for 254 children (121 boys and 133 girls).

Prior to each survey, all parents and guardians received printed information regarding the study, including the dose of radiation exposure from dual-energy X-ray absorptiometry (DXA) and signed a letter of consent. Children were allowed to decline participation on their own accord at any time. This study was performed in accordance with the ethical standards set forth in the Declaration of Helsinki and approved by the Ethics Committee of the Kindai University Faculty of Medicine.

### Bone area and mineral measurements

Measurements of whole-body bone area and bone mineral parameters were made using one DXA scanner (QDR-4500A, Hologic Inc., Bedford, MA, USA) mounted on a mobile examination car at each school. One experienced radiological technologist performed all scans and analyses at both baseline and follow-up surveys. The Step Phantom was used for quality control of the DXA scanner throughout the surveys. Subjects wore light clothing without metal or solid objects during the scanning process. Total body less head (TBLH) bone mineral content (BMC) and TBLH aBMD were measured in accordance with recommendations in the revised 2013 International Society for Clinical Densitometry Pediatric Official Positions [14]. The head region was separated by the neckline in the anterior scan image using standard manufacturer-recommended procedures [15]. Areal BMD (aBMD, g/cm^2^) was calculated by dividing BMC (g) by the projected area of bone (cm^2^). In addition, we calculated the bone mineral apparent density (BMAD), which accurately reflects changes in volumetric bone density during growth as an estimate of vBMD for children and adolescents, using the following formula: total body (TB) BMAD = TB BMC/(TB area^2^/body height) [16].

Abbreviations for bone parameters can be summarized as follows: TBLH BMC, total body less head bone mineral content (g); TBLH aBMD, total body less head areal bone mineral density (g/cm^2^); TB BMAD, total body apparent bone mineral density as an estimate of volumetric bone density (vBMD).

### Other variables

Bodyweight and height were measured in light clothing with no shoes. Height and weight were measured to the nearest 0.1 cm and 0.1 kg, respectively. Body mass index (kg/m^2^) was calculated by dividing body weight by height squared. Blood samples were obtained by vein puncture at the time of the baseline survey, allowed to clot, and centrifuged. All serum samples were stored at − 80 °C until analysis. Serum IGF-I levels were measured using a commercially available kit (Quantikine® ELISA, Human IGF-I Immunoassay, R&D Systems, Inc., Minneapolis, MN, USA). The intra- and inter-assay coefficients of variation were 0.8% and 2.0%, respectively. Serum 25-hydroxyvitamin D levels were measured using a radioimmunoassay (25-hydroxyvitamin D ^125^I RIA Kit; DiaSorin Inc., Stillwater, MN, USA), as previously described [17]. The intra- and inter-assay coefficients of variation were 5.2–9.5% and 6.3–10.8%, respectively. We used a self-reported questionnaire comprising multiple-choice single-answer questions to obtain information regarding pubic hair appearance and time spent doing sedentary activities, such as watching television, playing video games, and using a mobile phone and/or personal computer. Together with their parents/guardians, children chose only one of the predefined options for pubic hair appearance (third grade, fourth grade, fifth grade, or no appearance) and sedentary behavior (< 1 h/day, ≥ 1 and < 2 h/day, ≥ 2 and < 3 h/day, ≥ 3 and < 4 h/day, or ≥ 4 h/day).

### Statistical analysis

Differences between the study population and the dropout population were assessed using the unpaired *t* test. The unpaired *t* test or Mann-Whitney *U* test was used to assess sex difference at the baseline survey. The paired *t* test was used to assess differences between baseline and follow-up surveys. Pearson’s correlation coefficient was used to evaluate relationships of IGF-I levels with anthropometric variables and bone parameters. In addition, subjects were stratified by sex-specific baseline IGF-I levels (from IGF-I Q1, the lowest quartile (Q) group of IGF-I levels, to IGF-I Q4, the highest quartile group of IGF-I levels) before assessing relationships between IGF-I levels and bone variables. ANOVA was performed to explore differences among quartile groups of IGF-I levels. ANCOVA was used to explore differences among quartile groups of IGF-I levels after adjusting for confounding factors such as 25-hydroxyvitamin D, sedentary behavior, pubic hair appearance, weight, and height. To evaluate differences in bone mass at both baseline and follow-up among quartile groups, repeated measures ANOVA and repeated measures ANCOVA were performed. For trend tests on bone mineral parameters from the lowest to highest quartiles of IGF-I levels, multiple linear regression analysis was performed, with bone mineral parameters in each individual as the explanatory variable, and quartiles of IGF-I levels and confounding factors including 25-hydroxyvitamin D and others as dependent variables. *P* < 0.05 was considered statistically significant. All analyses were performed using SPSS Statistics Desktop for Japan, Version 22 (IBM Japan, Ltd., Tokyo, Japan).

## Results

Characteristics of the study population (follow-up subjects) and the dropout population are shown in Table 1. At baseline, there were no significant differences in anthropometric and bone variables between the study population and the dropout population. Serum levels of IGF-I in girls were significantly higher than those in boys at baseline. In addition, pubic hair in girls appeared significantly earlier than in boys. From baseline to follow-up, there were significant increases in height, weight, TBLH BMC, TB bone area, and TBLH aBMD in both boys and girls. In contrast, TB BMAD significantly decreased from baseline to follow-up.

**Table 1:** Characteristics of the study population and dropout population

	Study population							Dropout population			
	Baseline			Follow-up		^a^Baseline vs follow-up		Baseline		^b^Study vs dropout populations	
	Boys, *N* = 121	Girls, N=133	^b^Boys vs girls	Boys, *N* = 121	Girls, *N* = 133	Boys	Girls	Boys, *N* = 81	Girls, *N* = 73	Boys	Girls
Age (years)	11.2 ± 0.3	11.2 ± 0.3	ns	14.2 ± 0.3	14.2 ± 0.3	< 0.001	< 0.001	11.2 ± 0.3	11.2 ± 0.3	ns	ns
Height (cm)	141 ± 6	143 ± 7	0.007	162 ± 6	155 ± 5	< 0.001	< 0.001	142 ± 6	143 ± 7	ns	ns
Weight (kg)	34.7 ± 7.4	35.1 ± 5.9	ns	49.9 ± 8.4	47.0 ± 5.8	< 0.001	< 0.001	35.4 ± 7.1	35.5 ± 8.7	ns	ns
TBLH BMC (g)	644 ± 140	700 ± 162	0.003	1143 ± 251	1044 ± 180	< 0.001	< 0.001	656 ± 121	690 ± 178	ns	ns
TB bone area (cm^2^)	1288 ± 174	1336 ± 186	0.036	1789 ± 211	1680 ± 153	< 0.001	< 0.001	1305 ± 155	1329 ± 195	ns	ns
TBLH aBMD (g/cm^2^)	0.609 ± 0.040	0.624 ± 0.050	0.005	0.739 ± 0.074	0.720 ± 0.062	< 0.001	< 0.001	0.613 ± 0.037	0.618 ± 0.057	ns	ns
TB BMAD (g/cm^3^)	0.081 ± 0.008	0.080 ± 0.006	ns	0.075 ± 0.004	0.078 ± 0.005	< 0.001	0.003	0.081 ± 0.007	0.080 ± 0.007	ns	ns
25-hydroxyvitamin D (ng/mL)	34.3 ± 7.8	29.6 ± 7.7	< 0.001								
IGF-I (ng/mL)	198 ± 80	281 ± 81	< 0.001								
Sedentary behavior (media use), *N* (%)											
< 1 h/day	29 (24)	33 (25)	ns								
≥1, < 2 h/day	54 (45)	63 (47)									
≥ 2, < 3 h/day	20 (17)	29 (22)									
≥ 3, < 4 h/day	11 (9)	6 (5)									
≥ 4 h/day	7 (6)	2 (2)									
Pubic hair appearance, *N* (%)											
<Grade 5	0 (0)	10 (8)	< 0.001								
Grade 5	8 (7)	36 (27)									
No appearance	113 (93)	87 (65)									

*N* number, *ns* not significant, *TBLH* total body less head, *BMC* bone mineral content, *TB* total body, *aBMD* areal bone mineral density,

*BMAD* bone mineral apparent density as an estimate of volumetric bone mineral density, *IGF-I* insulin-like growth factor-I

Values represent mean ± SD or *N* (%)

^a^Paired *t* test was used to assess the difference between baseline and follow-up surveys

^b^*P* value calculated with unpaired *t* test or Mann-Whitney *U* test. *P* < 0.05 was considered statistically significant

Table 2 shows linear relationships of serum levels of IGF-I with anthropometric variables and bone mineral parameters. IGF-I levels showed significant positive relationships with height, weight, TBLH BMC, and TBLH aBMD in both sexes and at both baseline and follow-up. On the other hand, there were significant inverse relationships between IGF-I levels and TB BMAD in both sexes at baseline.

**Table 2:** Correlation coefficients between IGF-I levels at baseline and other variables

	Boys, *N* = 121		Girls, *N* = 133	
	*r*	*P*	*r*	*P*
Baseline				
Height	0.510	< 0.001	0.606	< 0.001
Weight	0.473	< 0.001	0.613	< 0.001
TBLH BMC	0.521	< 0.001	0.600	< 0.001
TB bone area	0.557	< 0.001	0.637	< 0.001
TBLH aBMD	0.371	< 0.001	0.512	< 0.001
TB BMAD	-0.435	< 0.001	-0.457	< 0.001
Follow-up				
Height	0.420	< 0.001	0.275	< 0.001
Weight	0.489	< 0.001	0.434	< 0.001
TBLH BMC	0.543	< 0.001	0.493	< 0.001
TB bone area	0.554	< 0.001	0.450	< 0.001
TBLH aBMD	0.455	< 0.001	0.461	< 0.001
TB BMAD	− 0.129	ns	0.140	ns

*IGF-I* insulin-like growth factor-I, *N* number, *TBLH* total body less head, *BMC*, bone mineral content, *TB* total body, *aBMD* areal bone mineral density, *BMAD* bone mineral apparent density as an estimate of volumetric bone mineral density, *ns* not significant

Pearson's correlation coefficient was used. *P* < 0.05 was considered statistically significant

Relationships between bone mineral parameters and quartile groups of IGF-I levels at baseline and follow-up are shown in Table 3. From the lowest to highest quartiles of baseline IGF-I levels, TBLH aBMD in boys showed a significant positive increase at follow-up after adjusting for confounding factors. In girls, TB BMAD at follow-up showed a significant positive increase from the lowest to highest quartiles of baseline IGF-I levels after adjusting for confounding factors.

**Table 3:** Relationships between bone mineral parameters and quartile groups of IGF-I levels

			Baseline					Follow-up					Baseline and follow-up	
			Mean	SD	*P* for ANOVA	*P* for ANCOVA	*P* for Trend	Mean	SD	*P* for ANOVA	*P* for ANCOVA	*P* for Trend	*P* for repeated measures ANOVA	*P* for repeated measures ANCOVA
Boys, *N* = 121	TBLH BMC (g)													
		IGF-I Q1	567	97	< 0.001	ns^a^	ns^a^	963	179	< 0.001	0.013^a^	ns^a^	< 0.001^a^	0.044^a^
		IGF-I Q2	607	88				1110	195					
		IGF-I Q3	654	139				1167	259					
		IGF-I Q4	746	159				1333	222					
	TBLH aBMD (g/cm^2^)													
		IGF-I Q1	0.594	0.038	0.003	ns^b^	ns^b^	0.692	0.059	< 0.001	0.011^b^	0.007^b^	< 0.001^b^	0.004^b^
		IGF-I Q2	0.603	0.030				0.740	0.072					
		IGF-I Q3	0.607	0.038				0.739	0.072					
		IGF-I Q4	0.630	0.046				0.786	0.066					
	TB BMAD (g/cm^3^)													
		IGF-I Q1	0.085	0.006	< 0.001	ns^b^	ns^b^	0.076	0.005	0.039	ns^b^	ns^b^	< 0.001^b^	ns^b^
		IGF-I Q2	0.083	0.006				0.077	0.004					
		IGF-I Q3	0.080	0.008				0.074	0.004					
		IGF-I Q4	0.077	0.008				0.075	0.004					
Girls, *N* = 133	TBLH BMC (g)													
		IGF-I Q1	562	113	< 0.001	ns^a^	ns^a^	918	156	< 0.001	ns^a^	ns^a^	< 0.001^a^	ns^a^
		IGF-I Q2	692	124				1042	159					
		IGF-I Q3	738	144				1090	165					
		IGF-I Q4	808	158				1125	176					
	TBLH aBMD (g/cm^2^)													
		IGF-I Q1	0.586	0.039	< 0.001	ns^b^	ns^b^	0.679	0.053	< 0.001	ns^b^	ns^b^	<0.001^b^	ns^b^
		IGF-I Q2	0.625	0.041				0.718	0.063					
		IGF-I Q3	0.634	0.043				0.734	0.056					
		IGF-I Q4	0.653	0.050				0.749	0.057					
	TB BMAD (g/cm^3^)													
		IGF-I Q1	0.083	0.006	< 0.001	ns^b^	ns^b^	0.077	0.005	ns^b^	0.036^b^	0.006^b^	ns^b^	ns^b^
		IGF-I Q2	0.080	0.005				0.078	0.004					
		IGF-I Q3	0.078	0.006				0.078	0.006					
		IGF-I Q4	0.077	0.005				0.079	0.004					

*IGF-I* insulin-like growth factor-I; *TBLH* total body less head, *BMC* bone mineral content, *N* number, *Q* quartile, *ns* not significant, *aBMD* areal bone mineral density, *TB* total body, *BMAD* bone mineral apparent density as an estimate of volumetric bone mineral density, *IGF-I Q1* the lowest quartile group of IGF-I levels, *IGF-I Q4* the highest quartile group of IGF-I levels

ANOVA and ANCOVA were performed to explore differences among quartiles

Multiple linear regression analysis was performed for trend tests from lowest to highest quartiles of IGF-I levels

Repeated measures ANOVA and ANCOVA were performed to explore differences among quartiles. *P* < 0.05 was considered statistically significant

^a^Adjusted for 25-hydroxyvitamin D, sedentary behavior, pubic hair appearance, weight, and height at baseline

^b^Adjusted for 25-hydroxyvitamin D, sedentary behavior, pubic hair appearance, and weight at baseline

Table 4 shows the relationships between changes in bone mineral parameters from baseline to follow-up and quartile groups of baseline IGF-I levels. In boys, there was a significant positive increase in changes in TBLH BMC from the lowest to highest quartiles of baseline IGF-I levels after adjusting for confounding factors, including height. Changes in TBLH aBMD also showed a significant positive increase after adjusting for confounding factors. In girls, there was a significant positive increase in changes in TB BMAD from the lowest to highest quartiles of baseline IGF-I levels after adjusting for confounding factors.

**Table 4:** Relationships between change in bone mineral parameters from baseline to follow-up and quartile groups of IGF-I levels

			Mean	SD	*P* for ANOVA	*P* for ANCOVA	*P* for trend
Boys, *N* = 121	Change in TBLH BMC (g)						
		IGF-I Q1	396	112	< 0.001	0.005^a^	0.002^a^
		IGF-I Q2	502	144			
		IGF-I Q3	514	158			
		IGF-I Q4	586	114			
	Change in TBLH aBMD (g/cm^2^)						
		IGF-I Q1	0.098	0.041	< 0.001	0.010^b^	0.012^b^
		IGF-I Q2	0.138	0.062			
		IGF-I Q3	0.132	0.056			
		IGF-I Q4	0.155	0.048			
	Change in TB BMAD (g/cm^3^)						
		IGF-I Q1	− 0.009	0.005	< 0.001	ns^b^	ns^b^
		IGF-I Q2	− 0.006	0.006			
		IGF-I Q3	− 0.006	0.007			
		IGF-I Q4	− 0.002	0.007			
Girls, *N* = 133	Change in TBLH BMC (g)						
		IGF-I Q1	356	90	ns	ns^a^	ns^a^
		IGF-I Q2	350	99			
		IGF-I Q3	352	106			
		IGF-I Q4	317	108			
	Change in TBLH aBMD (g/cm^2^)						
		IGF-I Q1	0.093	0.043	ns	ns^b^	ns^b^
		IGF-I Q2	0.093	0.049			
		IGF-I Q3	0.100	0.053			
		IGF-I Q4	0.096	0.046			
	Change in TB BMAD (g/cm^3^)						
		IGF-I Q1	− 0.007	0.006	< 0.001	ns^b^	0.017^b^
		IGF-I Q2	− 0.002	0.006			
		IGF-I Q3	0.000	0.006			
		IGF-I Q4	0.002	0.005			

*IGF-I* insulin-like growth factor-I, *TBLH* total body less head, *BMC* bone mineral content, *N* number, *Q* quartile, *aBMD* areal bone mineral density, TB total body, *BMAD* bone mineral apparent density as an estimate of volumetric bone mineral density, *ns* not significant, *IGF-I Q1* the lowest quartile group of IGF-I levels, *IGF-I Q4* the highest quartile group of IGF-I levels

ANOVA and ANCOVA were performed to explore differences among quartiles

Multiple linear regression analysis was performed for trend tests from the lowest to highest quartile of IGF-I

*P* < 0.05 was considered statistically significant

^a^Adjusted for 25-hydroxyvitamin D, sedentary behavior, pubic hair appearance, weight, and height at baseline

^b^Adjusted for 25-hydroxyvitamin D, sedentary behavior, pubic hair appearance, and weight at baseline

## Discussion

Mechanical loading stimulates skeletal growth, and the response to mechanical loading is a complex process regulated by multiple signaling pathways including that of IGF-I [18]. However, little has been reported regarding the relationship between serum levels of IGF-I and bone status during childhood in prospective cohort studies. The present prospective 3-year follow-up study of community-dwelling children showed that serum levels of IGF-I at baseline were related to the acquisition of BMC, aBMD, and estimated vBMD later on. Pubertal children who had high levels of baseline serum IGF-I tended to have high bone mass at follow-up. Mean serum IGF-I levels reportedly increase slowly in prepubertal children and increase steeply during puberty, and peak serum levels of IGF-I are observed in girls aged 14.5 years and in boys aged 15.5 years [19, 20]. According to previous reports, the mean age of peak calcium accretion is 14.0 years for boys and 12.5 years for girls [3]. In the present study, subjects were followed over the course of 3 years from 11 to 14 years of age, and our study population was near the age at which calcium accretion and levels of serum IGF-I are maximal. A prospective study that spanned up to 9 years by Breen et al. reported a significant positive relationship between changes in IGF-I levels and changes in BMC among 78 children aged 4–8 years at baseline [12]. On the other hand, a study with a relatively small sample size (*n* = 37) reported no significant association between IGF-I levels and gain in bone mass among children [11]. Along with these previous reports, our present findings suggest that serum levels of IGF-I in children undergoing skeletal growth spurt are associated with future bone mass acquisition.

In the baseline survey, IGF-I levels showed significant positive relationships with bone area, BMC, and aBMD in both sexes, and a significant inverse relationship with vBMD in both sexes. In addition, aBMD significantly increased from 11 to 14 years of age, while vBMD significantly decreased during the same period. aBMD (g/cm^2^) is calculated by dividing BMC (g) by the projected area of bone (cm^2^). On the other hand, vBMD is calculated using bone area and bone depth. Bone size and mineral content reportedly increase independently during puberty, with the increase in bone size being proportionally faster than the rate of mineralization, and vBMD falls as bone size increases during early puberty [21]. Other studies have reported that accrual of bone mass mainly results from an increase in bone size, with minor changes in vBMD during growth [22], and that vBMD remains almost constant during pubertal development, in contrast to the increase in aBMD during puberty [23, 24]. The normative reference for Thai children has reported that vBMD of the lumbar spine decreases from age 8–9 years to age 10–11 years in boys and from age 10–11 years to age 11–12 years in girls, while aBMD of the lumbar spine increases during the same periods in both sexes [25]. Consistent with this, we found significant decreases in vBMD from baseline to follow-up, and a significant increase in aBMD. We also found sex differences in the relationship between IGF-I levels and bone parameters, with a more prominent decrease in vBMD in boys than in girls. Furthermore, baseline IGF-I levels were related with BMC and aBMD at follow-up in boys and vBMD at follow-up in girls. In general, differences in biological age have been observed between boys and girls. At ages 11 and 14 years, boys are on average 1.5 to 2 years delayed in physical maturity. Consistent with this, the present study observed a significant difference in pubic hair appearance between boys and girls at age 11 years. In addition, maximal increase in bone variables occurs earlier in girls than in boys [21], and sex differences in bone microarchitecture, geometry, density, and strength from childhood to early adulthood have also been reported [26]. Differences in bone growth between boys and girls may account for the sex differences in the relationship between IGF-I levels and bone mineral parameters.

The present study has several strengths. First, this is the first population-based prospective cohort study on this topic, which found a significant positive relationship between baseline levels of serum IGF-I and bone mineral acquisition later on. Second, the present study reflects the health status of community-dwelling children in Japan. The temporal sequence of exposure (IGF-I levels at baseline) and outcome (bone parameters at follow-up) in the cohort design strengthens the process of inferring cause-effect relationships. Third, the study population was relatively large compared with those of similar studies on this topic [11, 12]. Fourth, the present study is a single-center cohort and thus is free from inter-center variation. Fifth, a single radiological technologist performed all scans and scan analyses using a single DXA scanner.

There are also several limitations worth noting. First, the study population consisted only of 62.3% of participants from the baseline survey. Participant dropout may have resulted in self-selection bias. However, there were no significant differences in anthropometric and bone variables between the study population and the dropout population, which limited the impact of self-selection bias. Second, we did not obtain information regarding the tanner scale, physical activity, or nutrient intake, which could be associated with IGF-I levels and bone mineral parameters.

In conclusion, the present population-based prospective cohort study found that serum levels of IGF-I at 11 years of age were associated with the acquisition of BMC, aBMD, and vBMD later on. These findings suggest that children with high levels of serum IGF-I tend to have high bone mass during pubertal development.

## Data Availability

Not applicable.

## Abbreviations

aBMD: Areal bone mineral density
BMAD: Bone mineral apparent density
BMC: Bone mineral content
DXA: Dual-energy X-ray absorptiometry
IGF-I: Insulin-like growth factor-I
Q: Quartile
TB: Total body
TBLH: Total body less head
vBMD: Volumetric bone mineral density
